# Research priorities in regional anaesthesia: an international Delphi study

**DOI:** 10.1016/j.bja.2024.01.033

**Published:** 2024-03-05

**Authors:** Jenny Ferry, Owen Lewis, James Lloyd, Kariem El-Boghdadly, Rachel Kearns, Eric Albrecht, Fernando Altermatt, Balakrishnan Ashokka, Amany E. Ayad, Ezzat S. Aziz, Lutful Aziz, Balavenkatasubramanian Jagannathan, Noreddine Bouarroudj, Ki Jinn Chin, Alain Delbos, Alex de Gracia, Vivian H.Y. Ip, Kwesi Kwofie, Sebastian Layera, Clara A. Lobo, Mohammed Mohammed, Eleni Moka, Milena Moreno, Bethan Morgan, Arthur Polela, Poupak Rahimzadeh, Suwimon Tangwiwat, Vishal Uppal, Marcelo Vaz Perez, Thomas Volk, Patrick B.Y. Wong, James S. Bowness, Alan J.R. Macfarlane

**Affiliations:** 1 Department of Anaesthesia, Aneurin Bevan University Health Board, Newport, South Wales, UK; 2 Department of Anaesthesia & Perioperative Medicine, Guy's and St Thomas' NHS Foundation Trust, London, UK; 3 Centre for Human and Applied Physiological Sciences, King's College London, London, UK; 4 Department of Anaesthesia, Glasgow Royal Infirmary, Glasgow, UK; 5 School of Medicine, Dentistry and Nursing, University of Glasgow, Glasgow, UK; 6 University Hospital of Lausanne, Lausanne, Switzerland; 7 Department of Anaesthesia, University of Lausanne, Lausanne, Switzerland; 8 Department of Anesthesiology, Escuela de Medicina, Pontificia Universidad Católica de Chile, Santiago, Chile; 9 National University Health System, Singapore, Singapore; 10 Department of Anesthesia, ICU and Pain, Cairo University, Cairo, Egypt; 11 Department of Anaesthesia and Pain Medicine, Evercare Hospital, Dhaka, Bangladesh; 12 Ganga Medical Centre and Hospital, Coimbatore, India; 13 Clinique Maissalyne Constantine, Constantine, Algeria; 14 Department of Anesthesiology and Pain Medicine, University of Toronto, Toronto, ON, Canada; 15 Department of Anesthesiology and Pain Medicine, Toronto Western Hospital, Toronto, ON, Canada; 16 Department of Anesthesia, Medipole Garonne, Toulouse, France; 17 Hospital Rafael Estevez, Caja de Seguro Social, Aguadulce, Panama; 18 Department of Anesthesia and Pain Medicine, University of Alberta Hospital, Edmonton, AB, Canada; 19 Department of Anesthesia, Pain Management and Perioperative Medicine, Dalhousie University, Halifax, NS, Canada; 20 Department of Anesthesiology and Perioperative Medicine, University of Chile, Santiago, Chile; 21 Cleveland Clinic Abu Dhabi, Abu Dhabi, UAE; 22 North Cumbria University Hospitals, Carlisle, UK; 23 Creta InterClinic Hospital, Hellenic Healthcare Group (HHG), Heraklion, Crete, Greece; 24 Department of Anaesthesiology, Pontifical Xavierian University, Bogotá, Colombia; 25 Hospital Universitario San Ignacio, Bogotá, Columbia; 26 Wythenshawe Hospital, Manchester University NHS Foundation Trust, Manchester, UK; 27 Department of Anaesthesia and Critical Care, Levy Mwanawasa University Teaching Hospital, Lusaka, Zambia; 28 Pain Research Center, Department of Anesthesiology, School of Medicine, Iran University of Medical Sciences, Tehran, Iran; 29 Department of Anesthesiology, Faculty of Medicine Siriraj Hospital, Mahidol University, Bangkok, Thailand; 30 Departament of Anesthesiology and Pain Therapy of Faculdade de Ciências Médicas da Santa Casa de São Paulo, São Paulo, Brazil; 31 Department of Anaesthesiology, Intensive Care and Pain Therapy, Saarland University Medical Centre, Homburg, Germany; 32 Faculty of Medicine, Saarland University, Homburg, Germany; 33 Department of Anesthesiology and Pain Medicine, University of Ottawa, Ottawa, ON, Canada; 34 Nuffield Department of Clinical Neuroscience, University of Oxford, Oxford, UK

**Keywords:** pain management, priority setting, regional anaesthesia, research, training and assessment

## Abstract

**Background:**

Regional anaesthesia use is growing worldwide, and there is an increasing emphasis on research in regional anaesthesia to improve patient outcomes. However, priorities for future study remain unclear. We therefore conducted an international research prioritisation exercise, setting the agenda for future investigators and funding bodies.

**Methods:**

We invited members of specialist regional anaesthesia societies from six continents to propose research questions that they felt were unanswered. These were consolidated into representative indicative questions, and a literature review was undertaken to determine if any indicative questions were already answered by published work. Unanswered indicative questions entered a three-round modified Delphi process, whereby 29 experts in regional anaesthesia (representing all participating specialist societies) rated each indicative question for inclusion on a final high priority shortlist. If ≥75% of participants rated an indicative question as ‘definitely’ include in any round, it was accepted. Indicative questions rated as ‘definitely’ or ‘probably’ by <50% of participants in any round were excluded. Retained indicative questions were further ranked based on the rating score in the final Delphi round. The final research priorities were ratified by the Delphi expert group.

**Results:**

There were 1318 responses from 516 people in the initial survey, from which 71 indicative questions were formed, of which 68 entered the modified Delphi process. Eleven ‘highest priority’ research questions were short listed, covering themes of pain management; training and assessment; clinical practice and efficacy; technology and equipment.

**Conclusions:**

We prioritised unanswered research questions in regional anaesthesia. These will inform a coordinated global research strategy for regional anaesthesia and direct investigators to address high-priority areas.

**Editor's key points**

• The practice of regional anaesthesia has seen rapid growth following the introduction of ultrasound-guided techniques, which is driving research to improve patient outcomes.
• This international research prioritisation exercise included 29 specialists from six regional anaesthesia societies who performed a three-round modified Delphi process and literature review, resulting in 11 highest priority research questions.
• These research priorities cover themes of pain management, training and assessment, clinical practice and efficacy, technology and equipment, and will inform a coordinated global research strategy for regional anaesthesia.

Much has changed during the evolution of regional anaesthesia practice since its introduction in the 19th century.^1^ Research, however, remains a constant and critical component of innovation in advancing patient care and, ultimately, the specialty. Recently, research has focused on the development of ultrasound-guided fascial plane blocks^2^^,^^3^ and advances in technology.^4^ As a result, the breadth and complexity of regional anaesthesia has increased. In contrast, Turbitt and colleagues^5^ suggested that the anaesthesia community should emphasise greater simplicity in regional anaesthesia practice, proposing high-value, safe, and effective ‘Plan A Blocks’ to enable clinician engagement and increase patient access to regional anaesthesia. Recent initiatives have attempted to build on this proposal through attempts to standardise and prioritise elements of clinical practice and teaching.^5^, ^6^, ^7^, ^8^, ^9^

No initiative currently exists to focus research efforts in regional anaesthesia.^10^ The James Lind Alliance (JLA) conducts priority setting partnerships in the UK, which attempt to ‘address the mismatch between what researchers want to research and the practical information that is needed day to day’.^11^ In 2015, the JLA published the top 10 overall research priorities for anaesthesia and perioperative care.^12^ Whilst some of these were indirectly linked to regional anaesthesia, this process did not specifically address regional anaesthesia or incorporate international perspective. We therefore performed an international modified Delphi exercise led by Regional Anaesthesia-UK (RA-UK), with input from an international group of experts representing five specialist regional anaesthesia societies, to form consensus and prioritise research in globally.

## Methods

### Organisations represented

This study was led by RA-UK on behalf of the participating specialist regional anaesthesia (RA) societies. The following societies were invited to participate in the study; American Society of Regional Anesthesia and Pain Medicine, African Society of Regional Anesthesia, the Asian and Oceanic Society of Regional Anesthesia and Pain Medicine, Canadian Anesthesiologists' Society Regional Anesthesia section, European Society of Regional Anaesthesia and Pain Therapy, and the Latin American Society of Regional Anesthesia. All participating societies endorse the methodology and results.

### Ethical approval

The Clinical Trials and Research Governance Team, Research Services, University of Oxford (Oxford, UK), advised that no ethical approval or research governance was required for this survey. This was confirmed using the UK Health Research Authority Decision Tool (Supplementary material A). This manuscript is presented in line with published guidance on reporting Delphi studies.^13^

### Initial survey

To gauge opinion of the worldwide anaesthesia community, participating societies canvassed their members to submit three regional anaesthesia-related research questions via a Microsoft Forms survey (Supplementary material B). An e-mail invitation was circulated by the societies to their membership, and was also advertised by members of the steering committee on X (formerly known as Twitter). Respondents were asked to submit basic demographic information alongside their questions to enable assessment of representation by geographical region. Participating societies sent periodic reminder e-mails to their memberships to achieve the maximum response. Where necessary, societies translated the survey text from English into their primary language and sent this alongside the invitation (all responses were translated back into English for analysis). The initial survey remained open from May 22, 2022 to August 12, 2022.

Regional Anaesthesia-UK had earlier commenced a multidisciplinary priority-setting partnership for the UK, with similar goals but focused on UK priorities. This UK project invited all members of the healthcare team who perform regional anaesthesia, look after patients receiving regional anaesthesia, and patients who have undergone regional anaesthesia. The initial UK survey (open from February 25, 2022 until May 27, 2022) asked participants to respond to the same question as above. Responses submitted to the UK project by anaesthetists (identified by self-reported demographics) were imported into the international data set for this study.

### Longlist of indicative questions

Responses were downloaded into Microsoft Excel (Redmond, WA, USA). Those requiring translation were identified and sent to interpreters nominated by the partner organisations. All entries were labelled with a number and letter to ensure that each individual free text response could be traced back to the original entry. Questions with identifiable characteristics (e.g. named individual or hospital) were anonymised.

All responses were then independently reviewed by two members of the data management team (JF and OL); any that did not directly relate to regional anaesthesia were deemed ‘out of scope’. Where both reviewers agreed, this response was removed from further analysis. Where there was disagreement, a third member of the data management team (JL) reviewed the response to adjudicate. The list of ‘out of scope’ responses was ratified by the steering committee (Supplementary material C) and not included for further analysis.

In a manner consistent with previous priority-setting partnerships,^14^^,^^15^ free text ‘in scope’ responses were grouped into themes depending on their content. Some responses were placed into more than one theme where appropriate. Indicative questions (IQs) were formed from the themed data, with the aim of presenting each theme in the form of one or more discrete research question. This process resulted in IQs with a broader scope than the original submitted research ideas, with the intention of creating research subjects rather than specific hypotheses. Research feasibility of the IQ was not considered as this would have added a further limitation to the written IQ, rather than accurately reflecting the submitted responses. There were no minimum or maximum numbers of responses required to create each IQ, and each IQ covered all components of the responses within it. Themes with strong similarity were combined into a single IQ, whereas themes that appeared broad were split into multiple separate IQs. All ‘in scope’ responses contributed to at least one IQ. Once formed, the IQs were ratified by the steering committee.

### Literature review

To ensure that IQs had not been already answered in existing published literature, we conducted a literature review. A medical librarian (BM) and data management team agreed a search strategy for each IQ, then performed the search on MEDLINE (OVID), Embase (OVID), The Cochrane Library (Wiley), CINHAL (EBSCO), and PsycINFO (ProQuest).

In line with JLA practice, results were limited to level one evidence published in English within the last 3 yearrs.^16^

The data management team (JF, OL, and JL) reviewed the literature to determine whether IQs had been answered. An IQ was considered fully answered if the literature found was deemed to thoroughly address all components of the IQ. Any additional literature felt to be significant by the steering committee, but not identified in the literature review process, was also analysed. The steering committee then reviewed and ratified this process. All fully answered IQs were removed from further analysis. All partly answered or unanswered IQs were then entered into a three-round modified Delphi process.

Recent Delphi projects in regional anaesthesia^6^^,^^7^ have followed the direction of Akins and colleagues^17^ who state that a relatively small number of experts can be used for a Delphi process provided they have similar training and understanding of the area being studied. Thus, each participating society was asked to select five experts for the Delphi process alongside the steering committee (excluding the data management team), giving a Delphi team of 29 members. A target response rate was set at 20 people for each Delphi round.

### Modified Delphi technique

A modified Delphi process was used,^18^ similar to that used in other recent regional anaesthesia Delphi projects (including rating scores and thresholds for acceptance).^6^, ^7^, ^8^ Each round was conducted anonymously and remotely using Microsoft Forms, to enable experts from the international community to contribute equally.

Experts were asked to review the long list of IQs with the following question stem: ‘*Should the [IQ] be included in the final shortlist?*’ The randomisation function of Microsoft forms was utilised to present the IQs in a different order to each expert, to reduce the risk of question fatigue influencing outcomes.

Experts were asked to rate whether each IQ should be added to the shortlist of priority areas for regional anaesthesia research. Rating was performed using a four-point Likert scale (1, definitely; 2, probably; 3, probably not; 4, definitely not). If ≥75% of responses for an IQ were ‘1, definitely’, that IQ was included in the final shortlist (and not rated again in further Delphi rounds). Any IQ rated as ‘3, probably not’ or ‘4, definitely not’ by at least 50% of responses was rejected from the shortlist and removed from further rating rounds. IQs that were neither accepted nor rejected were retained for the next round of rating. Experts were also able to submit free text answers at each stage. After each round was completed, anonymised results were shared with all team members, along with responses to any comments made. In total, three rounds were conducted.

At the end of the final Delphi round, retained IQs were further stratified. Categorical responses given by each expert in round 3 were converted to numerical scores (definitely = 1; probably = 2; probably not = 3; definitely not = 4). Scores from the 29 experts were summed for each IQ, enabling the investigators to rank retained IQs numerically, with a lower total score constituting a higher ranking. The top 10 IQs from this ranking process were added to the single IQ accepted by the Delphi process. All Delphi participants were then invited to a final roundtable meeting, at which the data were reviewed and the final ‘highest priority’ research questions were ratified.

## Results

### Initial survey

**Fig 1:**
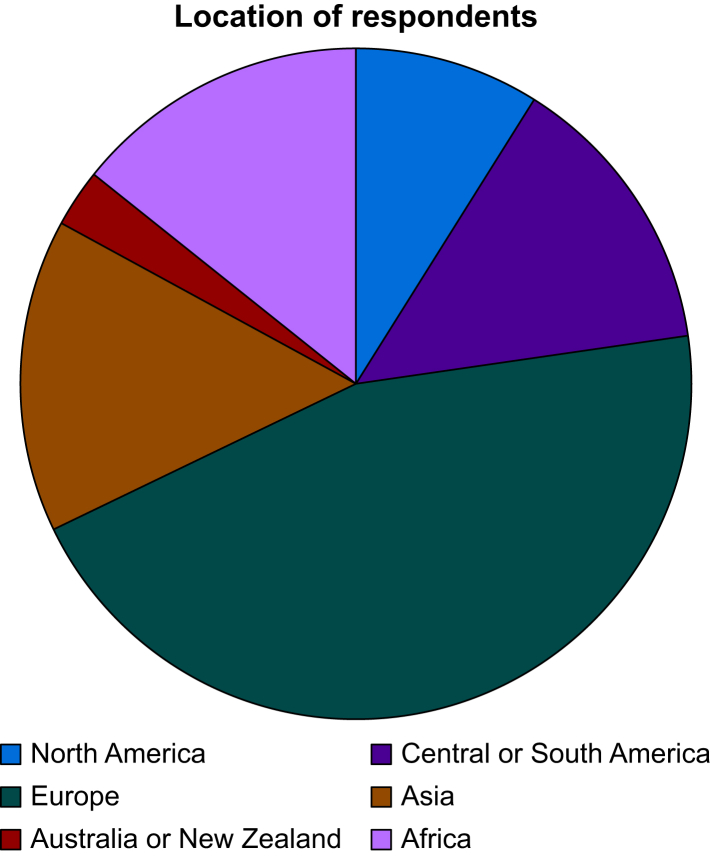
Pie chart showing location of respondents to initial survey.

All societies, with the exception of the American Society of Regional Anesthesia and Pain Medicine, accepted the invitation to participate in this study. In total, 516 people responded to the initial survey, providing 1318 free text responses. No duplicate questions were identified; 119 (9.0%) responses were deemed ‘out of scope’ and excluded from further analysis (Supplementary material C provides the full list of ‘out of scope’ responses). All participating societies were represented in the initial survey responses, with anaesthetists in Europe providing the greatest proportion of responses. A breakdown of respondents by location is provided in Fig 1.

Free text responses were condensed into 71 IQs (Supplementary material D). We reviewed 3485 papers as part of the literature review process, after which three IQs were deemed to be fully answered, leaving 68 IQs for the modified Delphi process.

Each round of the modified Delphi process was completed by all 29 expert members (100%). After Round 1, one IQ failed to meet the criteria to be retained and was excluded, leaving 67 IQs. In Round 2, one further IQ was excluded, thus 66 IQs entered Round 3. In the final round, one IQ met the criteria for inclusion, 63 IQs were retained, and two IQs were excluded.

**Fig 2:**
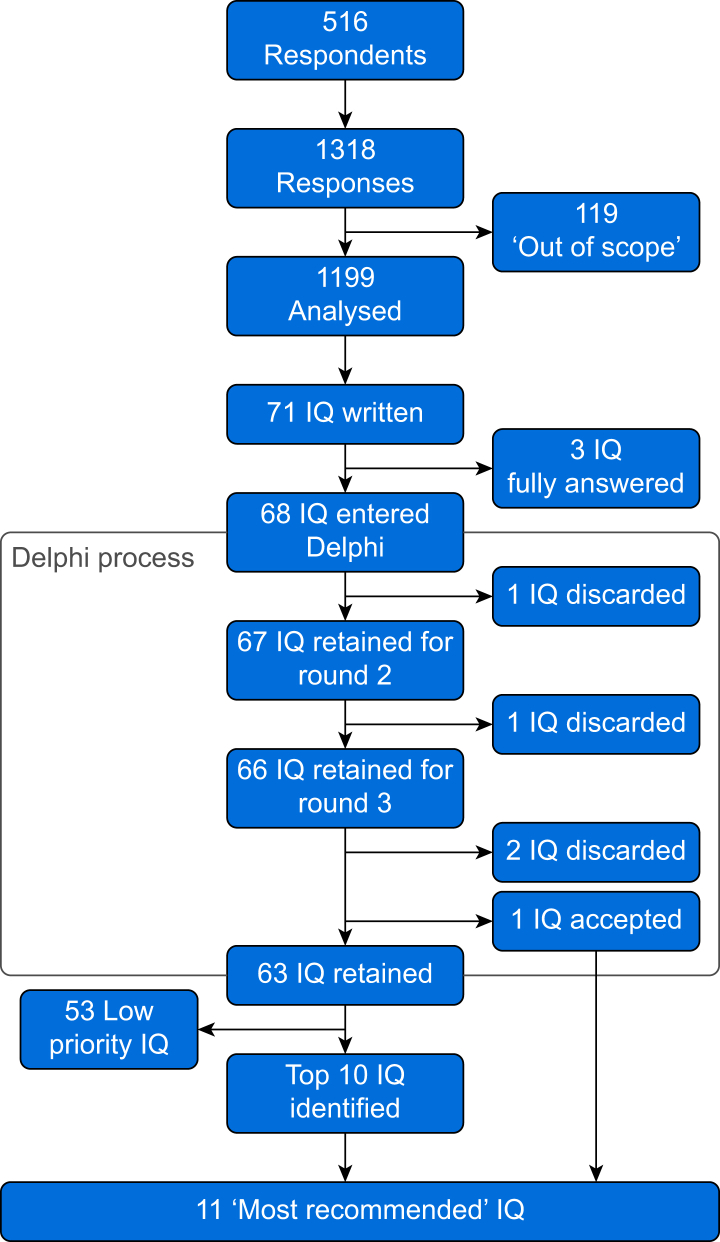
Flow chart showing data progress over study. IQ, indicative question.

The top 10 IQs from the numerical ranking process, added to the one accepted IQ from the modified Delphi process, contributed to the 11 ‘highest priority’ IQs shown in Table 1. Figure 2 shows data progress through the stages of the study.

**Table 1:** The 11 highest priority research questions identified in regional anaesthesia.

Indicative question	Theme
How can we best manage pain as regional anaesthesia wears off?	Pain management
What is the most effective way of delivering regional anaesthesia training?	Training and assessment
Can regional anaesthesia reduce chronic postsurgical pain?	Pain management
What is the clinical effectiveness of fascial plane blocks?	Conduct and efficacy
Can regional anaesthesia reduce long-term opioid use?	Pain management
What are the risks and benefits of using adjuncts to local anaesthetics?	Conduct and efficacy
How can novel technologies improve regional anaesthesia?	Technology and equipment
How should competency in regional anaesthesia be demonstrated?	Training and assessment
Does regional anaesthesia increase the risk of harm from compartment syndrome?	Conduct and efficacy
How can regional anaesthesia be used most effectively for trauma patients?	Conduct and efficacy
What role does regional anaesthesia have in the management of patients with chronic pain?	Pain management

## Discussion

### Summary

This study presents a list of 11 ‘highest priority’ research questions in regional anaesthesia identified and agreed by an international group of experts. By the nature of the process of forming the indicative questions, each one is broad based and not necessarily answered by a single research project. This approach is consistent with other published research priority setting exercises.^14^^,^^15^^,^^19^ We believe this will be an important resource to inform the immediate international regional anaesthesia research agenda. We hope it will help further develop clinical practice by guiding anaesthetists who engage in regional anaesthesia research and help bring the benefits of high-quality regional anaesthesia to a greater number of patients worldwide.

### Analysis of themes

Whilst the 11 ‘highest priority’ IQs encompass a diverse range of subjects in regional anaesthesia, four key themes are evident: pain management; training and assessment; clinical practice and efficacy; technology and equipment.

#### Pain management

The only IQ accepted by the modified Delphi process concerned the management of pain as regional anaesthesia effects resolve; rebound pain is a recognised phenomenon occurring in up to 40% of patients as the block resolves and can be challenging to treat.^20^, ^21^, ^22^ Work by Barry and colleagues^23^ identified a number of risk factors for development of rebound pain, highlighting the need for further research into this phenomenon.

Alongside the IQ on rebound pain, pain management is a theme in three other ‘highest priority’ IQs. One IQ concerns the need for opioid-sparing approaches to analgesia, which is particularly pertinent in light of the current opioid epidemic.^24^ Regional anaesthesia can be used as an alternative to, or in addition to, systemic analgesia and the benefits are not confined to the acute perioperative period (e.g. analgesia for rib fractures). This is an important area of investigation in order to maximise the utility of these techniques. Two ‘highest priority’ IQs concern the management of chronic pain. Some, though not all, evidence supports the hypothesis that effective control of acute pain in certain procedures can reduce the risk of developing chronic pain.^25^^,^^26^ More work is needed to investigate this conjecture with respect to specific patient groups, surgical procedures and pain trajectory,^27^ individual regional techniques (including single-shot *vs* continuous infusions), and the overall role of regional anaesthesia in managing ongoing chronic pain.

#### Training and assessment

It is difficult to define a standard or best practice in regional anaesthesia training and assessment. Training varies between geographical regions depending on the clinical environment and availability of resources. Further work must be done to better understand the optimal approach, as highlighted by one ‘highest priority’ IQ. Another relates to assessment of competency. Current metrics for assessment to ensure performance standards in a clinical workforce are predominantly based upon subjective observation and feedback.^28^^,^^29^ Objective metrics, such as eye tracking^30^^,^^31^ and technology to calculate needle tip visibility^32^ have been used in research but are still in their infancy with respect to widespread utilisation. Further defining these parameters, and ensuring that all practitioners are trained to a high level and can achieve and maintain competence, will improve access to high-quality, safe regional anaesthesia for patients. This aligns with the work of Chuan and Ramlogan^33^ who recently published a diverse list of research topics for education in regional anaesthesia, including simulation, curriculum, knowledge translation, methodology, and assessment.

#### Clinical practice and efficacy

Performing a peripheral nerve block is a complex and multifaceted skill. Furthermore, regional anaesthesia incorporates many techniques, often performed in different ways for varying indications. Choosing the most appropriate block for a particular procedure in an individual patient is essential.^5^ Fascial plane blocks have recently gained popularity, though a full understanding of their mechanism of action, optimal indications, and clinical benefit is still lacking.^34^ A strong evidence basis for how blocks are performed is also essential; factors such as drug choice and volume need to be considered. Regional anaesthesia can be performed as a single-shot procedure, a continuous infusion or as intermittent boluses; deciding which to do, and when, requires careful thought. The use of adjuncts, which links to the IQ regarding managing pain as regional anaesthesia effect regresses, is highlighted in the ‘highest priority’ IQs. Whilst the evidence for adjuncts in peripheral nerve blocks is probably greatest for dexamethasone, best practice remains unclear.^22^^,^^35^ Finally, the clinical setting is equally important, as highlighted by two ‘highest priority’ IQs which identified the use of regional anaesthesia in trauma. Whilst regional anaesthesia can be of significant patient benefit in this area,^36^ debate continues around the potential added risk in certain situations, such as the use of regional anaesthesia in compartment syndrome.^37^

#### Technology and equipment

Ultrasound-guided regional anaesthesia has been described as the most important advance in regional anaesthesia of the new millennium.^38^ It has been shown to increase block efficacy, decrease onset time, allow lower volumes of local anaesthetic to be used, and to reduce complications of vascular trauma and local anaesthetic toxicity.^38^ Novel technology has therefore facilitated some of the greatest progress in regional anaesthesia. As the world undergoes the fourth industrial revolution,^39^ artificial intelligence may be the most fundamental technology enabling change in how healthcare is delivered. It has the potential to become the next paradigm shift in regional anaesthesia^4^ and has already shown promise in supporting ultrasound scanning for regional anaesthesia.^40^, ^41^, ^42^ In addition, whilst augmented and virtual reality technologies offer the chance to support, improve, and reform traditional education and training in regional anaesthesia, more evidence is required to establish the potential role of these emerging tools.^32^^,^^42^, ^43^, ^44^

### Future directions

Although there is a wealth of published research in regional anaesthesia,^45^ with many studies ongoing, the scope at present is highly heterogeneous. Whilst such diversity will undoubtedly offer other insights that these ‘highest priority’ IQs do not, a coordinated approach for research into the clinical practice of regional anaesthesia will arguably hasten progress in the field, focusing on the topics that international experts have deemed most important. Minimising discrepancies can improve consistency of research and training, and ultimately improve delivery of regional anaesthesia to patients globally. Researchers, publishers, and funding bodies should therefore be aware of the priority areas identified in this work.

### Limitations

Despite the wide dissemination of our survey, there is a risk of responder bias, as relying on specialist society membership is likely to emphasise participation by regional anaesthesia enthusiasts. Not every regional anaesthesia society was able to participate in this project and Europe is overrepresented by respondents compared with population size. However, we did ensure representation from a wide range of healthcare settings globally and by participants of specialist societies from each continent. We did not include other healthcare workers or patients, who might prioritise different research questions because of their unique perspectives and lived experiences. This was a pragmatic choice, as coordinating input from other healthcare workers, patients, and the wider multidisciplinary team in the context of multiple languages is highly complex and beyond the scope of this study. These views are important, and a priority-setting partnership exercise is underway in the UK for this purpose. We did not stratify research priorities by specialist society, and the ‘highest priority’ IQs produced do not differentiate between different geographic regions (e.g. resource-poor environments could have differing priorities from the resource-rich). However, the initial survey and Delphi rating process considered the views of a geographically diverse population. IQs not included in the ‘highest priority’ list are themselves worthy of investigation (Supplementary material E). Finally, it is likely that this exercise will need to be repeated at periodic intervals (e.g. every 10 yr). When some of the ‘highest priority’ IQs are answered and new developments occur in the field, other priorities will doubtless emerge.

### Conclusions

We performed an open-access, large-scale survey of anaesthetists' opinions, ensuring representation from a range of income settings. We identified a shortlist of the highest priority research questions for the international regional anaesthesia community, as chosen by anaesthetists. Our findings will guide future research efforts in regional anaesthesia; inclusion on this list demonstrates that a question is of international importance and is worthy of allocating resources. These priorities are intended to underpin the development of a coordinated global research strategy in regional anaesthesia, accelerate scientific progress, and deliver benefit to patients worldwide.

## Authors’ contributions

Study concept, design, and conduct: JSB, JF, OL, JL, RK, KEB, AJRM

Data collection: all authors

Data analysis: JF, JSB

Manuscript preparation: JF, JSB

Manuscript editing: JF, JSB, RK, AJRM, KEB

Manuscript review and approval: all authors

## Acknowledgements

We thank the medical librarian team at Manchester NHS Foundation Trust for their help with the literature reviews performed as part of this study.

## Declarations of interest

JF and RJK are board members of Regional Anaesthesia UK. JF declares honoraria from Intelligent Ultrasound. KE-B or his institution has received funding from GE Healthcare, PAION, Fisher and Paykel, and Edwards Lifesciences. BA, JB, ST, PR, and LA are executive board members of the Asia Oceanic Society of Regional Anaesthesia and Pain Medicine. ESA is president and AA is general secretary of the African Society of Regional Anaesthesia. VHYI is vice chair and VU is chair of the Regional Anaesthesia section of the Canadian Anesthesiologists' Society. EA, EM, TV, and AJRM are members of the executive board of the European Society of Regional Anaesthesia & Pain Therapy. EM declares honoraria/lecture fees from Menarini. MM declares honoraria/lecture fees from the Aesculap Academy, B. Braun. MVP is an executive board of Latin American Society of Regional Anesthesia. TV declares research grants from Sedana Medical, Ratiopharm, Saarland, Pfizer, Infecto Pharm, and Cyto Sorbents Europe GmbH, and lecture fees from Pajunk and CSL Behring. AJRM is the current president of Regional Anaesthesia UK, and declares honoraria from Intelligent Ultrasound and GE Healthcare. JSB declares honoraria, research funding, or both from Intelligent Ultrasound.

## Funding

This study is part of the research and education strategy of Regional Anaesthesia UK (RA-UK). Publications arising may therefore present information as guidance from RA-UK. No funding declared.
