# Supplementary material for: Research priorities in regional anaesthesia: an international Delphi study
Source: Br J Anaesth. 2024 Mar 5;132(5):1041–8. doi: 10.1016/j.bja.2024.01.033 (PMC11103078; doi:10.1016/j.bja.2024.01.033)
Supplement: Multimedia component 2 [file mmc2.pdf]

Priority Research Areas in Regional Anaesthesia; an International Delphi Study

Introduction
Thank you for taking the time to complete this survey.

Why are we contacting you?
We want to find out what research is needed to improve the care of patients undergoing regional anaesthesia. We really want to hear ideas from regional anaesthetists, to help guide research in the direction you feel is most important.

What do you need to do?
Tell us any questions you have about regional anaesthesia that you would like to see answered by research. There are no right or wrong answers and the most important thing is to focus on what is important to you. We will also ask a little bit about you, so we can make sure we're collecting opinions from anaesthetists around the world. Finally, we'll ask for your consent to use the information you provide. The survey will take less than 10 minutes to complete. All personal information will be anonymised so it will not be possible to tell who submitted any of the answers.

The kind of input we need
The questions you suggest could be related to your own experience, there are no right or wrong suggestions, and we would value any input about areas you would like more information about.
Example questions from a James Lind Alliance Priority Setting Partnership entitled Anaesthesia and Perioperative Care included:

- How can patient care around the time of emergency surgery be improved?
- How can pre-operative exercise or fitness training, including physiotherapy, improve outcomes after surgery?
- How can we improve communication between the teams looking after patients throughout their surgical journey?

What we are hoping to find are some questions that haven't yet been researched or have not been researched enough. Your input is vital to ensure we identify the most important research questions to improve patient care.

1. Please enter your first question below:

2. Please enter your second question below:

3. Please enter your third question below:

4. About you, and what happens next

We need to know a bit about you so that we can make sure we're getting responses from a wide range of people. Please tell us a little bit about yourself through the following questions. This information will be treated confidentially: no-one will know what questions you proposed.

Which of the following is recorded on your medical record

- ◯ Male
- ◯ Female
- ◯ Other
- ◯ Not Specifiec
- ◯ I do not know

5. Where do you practice?

- ◯ North America
- ◯ Central or South America
- ◯ Europe
- ◯ Asia
- ◯ Australia or New Zealand
- ◯ Africa

This content is neither created nor endorsed by Microsoft. The data you submit will be sent to the form owner.

Microsoft Forms
