# Supplementary material for: Research priorities in regional anaesthesia: an international Delphi study
Source: Br J Anaesth. 2024 Mar 5;132(5):1041–8. doi: 10.1016/j.bja.2024.01.033 (PMC11103078; doi:10.1016/j.bja.2024.01.033)
Supplement: Multimedia component 3 [file mmc3.pdf]

Research priorities in regional anaesthesia: an international Delphi study

Supplementary material C - Out of scope free text responses

016c	A pratical analysis of the time : more time before the intervention for a shorter course after ?
023c	How does the risk of aspiration pneumonitis or pneumonia compare between deep sedation and GA (LMA or ETT can be grouped)?
026a	Is this something that will change practice in a positive way
026c	Is this not a duplication of previous research
032b	What conflicts of interest pertain to ultrasound technology changes and research grants. That is, when a newer machine is introduced, can individual researchers demonstrate honest distance between their promotion and the corporate promotion.
050a	What are the mechanisms of opioid tolerance and how can this be reversed?
057a	How can we improve care of the elderle patients in the preoperative period
057c	How can we develop pathways to standardised care of patients going to emergency surgery
077c	Is evidence based medicine better than personaliced medical care?
079a	What is the optimal and safe use of ischemia in orthopedic surgery?
079b	With the information already gathered in the literature, can we predict the risk of chronic postoperative pain for each patient?
080c	How to improve topical anesthesia?
085a	What's the main purpose of doing this researcher
102b	Tackling of the cultural interferance of labor pain management in many African countries.
112a	how can we manage the Iv fluids during emergency?
114a	Relation between prominent Adam's apple and difficult intubation
114b	Amnesia among anaesthesia doctors in theaters with non scavenging system
121a	What is the impact of long term lung function abnormalities on physical assessment scoring systems such as ASA
121b	What is the impact of acute and long term effects of Covid on the lungs on lung protective mechanical ventilation strategies
126b	As perioperative physicians preop and postop followup benifits to be enlightened. Most new coming r taking lightly.
133b	How to integrate acute and chronic pain ? not all acute pain physicians are chronic pain experts , more research to establish need for appropriate training and develop pathway for training which integrate both with application in acute settings
133c	How Lifestyle management measures can be used in treatment of acute and chronic pain practically
135b	Open excess to anatomy and 3 D photos with videos on website.
138c	For a successful Perioperative pain relief, surgeons and paramedical play an important role. Should we have more combined meetings.
141c	How good are the alternative forms of analgesic techniques in emergency situation
145a	(anonymised)
145b	(anonymised)
145c	(anonymised)
153a	Your opinion on regional anaesthesia?<br>(a)new concept (b) old concept with a new approach.<br>Ans b
154a	(anonymised)

154b	(anonymised)
154c	(anonymised)
155a	participation in a research study
155b	I want to participate in the research study
155c	research study
158c	Is role of Ketamine for pain relief more recognised today
161c	Anesthesiologists are not enough and other materials and appareils are not enough
163c	How to get involved into the developlment process on a such basic modular course as previously mentioned?
166a	(anonymised)
167a	(anonymised)
169a	What kind of anesthesia do you use in your hospital?
169b	Why do you use check-list before anesthesia?
184a	Preoperative assessment of patient health care and post operative problems.
193a	What if patient is made unaware of the regional anesthesia procedure..??<br>Like giving anaesthesia to provide regional anesthesia....?? Ofcourse after obtaining consent from the patient for the same..
193b	Why should we keep the arm abducted always after CNB....y not keepit in neutral position with extension lines always....
193c	Why can't we give spinal, for laparoscopic surgeries.....before GA........ r......... proceed solely under regional epispinal......
196a	Optimisation of airways and hemodynamics<br>Multimodal Analgesia preop, intraop and postoperative
196b	Properative fitness and exercise maintains control over autonomic system, cardio respiratory stability and good exercise tolerance of our body .<br>So hemodynamic stability maintained intraopwratively. It improves postoperative recovery , mobilisation of patient
196c	Communicate with teams is through WHO checklist ,<br>Communicate with surgeon regarding the surgical events anticipated and ongoing , blood losses any critical steps<br>Communicate with the staff for assistance ,difficult airway ,emergency drug kits,LAST kit and assistance available
198a	(anonymised)
205c	In public hospitals: Can a dedicated peri operative team of a physiotherapist, anesthetist and orthopaedic surgeon reduce hospital length of stay and time to functional ability, in post knee replacement patients.
211a	After assessment take a few minutes to yourself focussing on the plan of anesthesia n perioperative needs of the patient.<br>Communicate with the surgical team your plan n the perioperative needs you expect.<br>In case of smaller setups like mine communicate with the primary care physician and try to get him around(he is the busiest n takes time to get,. This allows you more time to prepare)
211b	Extremely beneficial. Respiratory physiotherapy being the most important. Physiotherapy can prevent DVT in a great way. Am not going into specific physiotherapy needs of individual surgery
211c	Always it's you who should bring change first. Be in good communication with the surgical, intensive care and most importantly primary care physician at all levels. Listening is a better way of communication

213a	How patients diet and nutritional status / gut microbiome markers can influence the perioperative outcome?
213b	Effect of neuromodulation in acute pain set up and which modality of neuromodulation provides better prolonged analgesia
213c	Effect of non invasive vagus stimulation on perioperative fitness improvement and outcome
215b	Inspite of good number of analgesics & sedatives, patient is still uncomfortable with pain, can't we sedate them for first 24 hours to lessen the degree and intensity of pain ?
225a	How can we know if the different participants in the evaluation of the patient's postoperative pain (doctors, nurses, technicians) are well trained to apply the visual analog scale?
225b	How can we know if the patient is well trained to apply the visual analog scale? Does the patient really understands the question?
229c	Is there any sedation and analgesia technique to identify the radiofrequency target regions that can be used to perform the procedures without the patients suffering so much?
230a	How important is it to keep OR temperatures low (<20 degrees celsius) for the prevention of infections in orthopedic surgery, especially in total joint replacement surgery?
230c	Program of preoperative talks about elective surgery to treat fear and anxiety in patients, to analyze its correlation with improved well-being and fewer analgesic requirements in the postoperative period
242a	How can we establish a pain clinic in low resource country?
244b	How can we built a multidisciplinary team for perioperative pain management?
244c	How can prehabilitation improve outcome? Which exercise should we choose, for which patient?
272a	How can improve US guided (peripheral) vascular access patient satisfaction in contrast with "classic" multiple attempts to place iv lines.
276c	Cryoneurolysis - is it an alternative for chronic pain patients and for acute pain management?
343a	Does the use of cerebral oximetry in beach chair position (mostly shoulder surgery) have any impact on immediate recovery (PONV, cognitive dysfunction) and reduction in the incidence of major complications like CVA's (like stroke)
373a	Main interest is obstetric
394c	Is ropivacaine available to use in this trust?
400a	Best (evidence based) way to teach POCUS to trainees
402c	national wide guidelines for paediatric pain departments
413b	Can measuring a continuous real time pH or lactate like oxygen saturation and ETCo2 make a difference in major surgical outcomes ?
413c	How anaesthetist should deal with unethical surgeon ?
416b	How can we improve communication between the teams looking after patients throughout their surgical journey?
420c	What is the most effective non-pharmacologic method (EMDR, VR, anti-catastrophizing training) to decrease the incidence of CPSP?
426b	What is the hard evidence, regarding costs, supporting the shift toward outpatient services in the orthopedic surgery setting?
431a	Yes
431b	Yes
431c	Yes
433a	(untranslateable)
433b	(untranslateable)

433c	(untranslateable)
434c	(untranslateable)
436c	What is the optimal complementary analgesia for outpatient pain management?
439b	In terms of burnout we should receive more leave of abscence days in addition to vacations.
439c	We should have comissions inside the regions to seek solutions to this problem
440a	Should I use gabapentinoids in the days prior to surgery to prevent postoperative pain after spinal surgery?
440b	Should I use gabapentinoids in the days prior to surgery to prevent neuropathic pain after liposculture surgery?
441c	Uses of POCUS pediatric anesthesia
446a	How can we improve patient care during emergency surgery?
446b	Can preoperative exercise, including physiotherapy, improve postoperative outcomes?
446c	How can we improve communication between the different teams caring for patients along their surgical journey?
450a	Which are the most important barriers for an optimal patient perioperative care?
450c	Do we need new guidelines for acute postoperative pain management?
461a	Care on hypotension
461b	Care on Pre loading of pregnant women
467a	Is perioperative genicular nerve radiofrequency effective to decrease pain after knee artroplasty?
478b	what connection should there be between the team that prepares the patient for surgery and that operates and that will take care after the operation?
479b	Does the opioid epidemic in west can extrapolate to opioid free anaesthesia world over, should be a multicontinental study.
486a	What are the role of intravenous lignocaine in patients recovery and outcome after cardiovascular and thoracic surgeries?
486c	How can we improve patient's perception about perioperative physicians role in improving overall outcome following cardiovascular and thoracic surgery?
489a	Why in the official journal there's no more case reports or brief publication's about specific clinical experiences.
493a	With regional anesthesia
495b	Does Pocus training of residents improve post op care and patient outcomes ,what percentage
496a	Why can we do a common questionnaire with the surgical teams which will be according to ERAS protocols in order to improve communication between the teams, so we will know what they ask and they will know what we ask?
496b	How the acute pain service and the physiotherapist can work together ?
500a	How to optimise morbitity and mortality in high risk patients receiving general anesthesia (strategies or guidelines)
500b	Who should be in charge for immediate postoperative care; surgical team or anesthetist? Does it make a difference on outcome?
504c	More data on gastric ultrasound in assesment of empty stomac.
508b	When patients undergoing surgery receive prehabilitation, can you achieve better pain tolerance and accelerated recovery, resuming daily and physical activities faster, decreasing the probability of comorbidities associated with prolonged immobility such as pulmonary thromboembolism?
508c	Making all parties involved in patient management know the objectives and the need to center the treatment in the patient and not in the disease

513b	I appreciate to tell me the role of transalting medicine as a part or through pain medicine, as a tough question?
513c	In the sfere odf molecular medicine there a lot of areas about genomics in pain medicine<br>I hope you understand and pay attention, thanks
514a	How to make sure all the patients receive their pre medication for day case?
514c	What extent PACU and ward team involve with day cases sending home? Any thing we could improve?
520a	Metaanalysis, Trial Sequential Analysis.... How statistics affect interpretation of clinical questions?
520b	From statistical significance to clinical relevance... Are we any smarter?
