# Supplementary material for: Research priorities in regional anaesthesia: an international Delphi study
Source: Br J Anaesth. 2024 Mar 5;132(5):1041–8. doi: 10.1016/j.bja.2024.01.033 (PMC11103078; doi:10.1016/j.bja.2024.01.033)
Supplement: Multimedia component 4 [file mmc4.pdf]

Research priorities in regional anaesthesia: an international Delphi study

Supplementary material D- Long list of IQ, with results of literature search

Indicative Question	Is this IQ answered?
How does sedation or general anaesthesia prior to block performance impact the safety of regional anaesthesia?	Not answered
Who should perform regional anaesthesia?	Partly answered
How can regional anaesthesia be used most effectively in hip surgery?	Partly answered
How can regional anaesthesia for head and neck procedures be improved?	Partly answered
What is the clinical effectiveness of fascial plane blocks?	Partly answered
How can the risk of adverse reactions to local anaesthetic be reduced?	Partly answered
How do the risks and benefits of regional anaesthesia change in different patient groups?	Partly answered
How can the safety of regional anaesthesia catheter techniques be ensured?	Partly answered
What are the risks and benefits of regional anaesthesia for children?	Partly answered
How can regional anaesthesia be used most effectively in abdominal surgery?	Partly answered
How can regional anaesthesia be used most effectively in breast surgery?	Partly answered
How can regional anaesthesia for emergency surgery be improved?	Not answered
How can patient experience of regional anaesthesia be improved?	Partly answered
Can regional anaesthesia reduce long term opioid use?	Not answered
How can regional anaesthesia be used most effectively for the intensive care patient?	Not answered
How can novel technologies improve regional anaesthesia?	Partly answered
How should competency in regional anaesthesia be demonstrated?	Partly answered
How can regional anaesthesia be used most effectively in elbow surgery?	Not answered
What is the role of nerve stimulators in ultrasound guided regional anaesthesia?	Partly answered
How can regional anaesthesia be used most effectively for chest wall trauma?	Partly answered

How can the effectiveness of regional anaesthesia be improved?	Partly answered
How can regional anaesthesia be used most effectively for a fractured neck of femur?	Partly answered
Does systemic absorption of local anaesthetic contribute to analgesia?	Partly answered
How can regional anaesthesia be improved in low resource environments?	Not answered
How can regional anaesthesia be used most effectively in knee surgery?	Partly answered
Which factors alter the duration of regional anaesthesia?	Partly answered
How often do complications occur as a result of regional anaesthesia?	Partly answered
How can the safety profile of regional anaesthesia be improved?	Partly answered
What is the safety profile of regional anaesthesia in patients with altered coagulation?	Partly answered
How can we improve the consent process for regional anaesthesia?	Partly answered
Does regional anaesthesia have an effect on cancer outcomes?	Not answered
How can regional anaesthesia be used most effectively in obstetric patients?	Partly answered
What is the optimal infusion regime for regional anaesthesia catheter techniques?	Partly answered
Can regional anaesthesia improve the management of pain after surgery?	Partly answered
How can regional anaesthesia be used most effectively in cardiothoracic surgery?	Partly answered
Can regional anaesthesia reduce opioid use in the perioperative period?	**FULLY ANSWERED**
How can regional anaesthesia be used to optimise operating theatre efficiency?	Partly answered
Can regional anaesthesia help to reduce the environmental impact of anaesthesia?	Not answered
Which, if any, sedation method should be used with regional anaesthesia?	Partly answered
How can regional anaesthesia be used most effectively in spinal surgery?	Partly answered
Can regional anaesthesia improve long term outcomes after surgery?	Partly answered
Can regional anaesthesia reduce chronic post surgical pain?	Partly answered

How should complications from regional anaesthesia be managed?	Partly answered
Is regional anaesthesia more cost effective than alternatives?	Partly answered
Is regional anaesthesia safer than general anaesthesia?	Partly answered
Does the safe dose of local anaesthetic vary between different patients and regional anaesthesia techniques?	Not answered
What are the risks and benefits of using adjuncts to local anaesthetics?	Partly answered
What is the most effective way of delivering regional anaesthesia training?	Partly answered
Which patients benefit most from regional anaesthesia?	Partly answered
How can we improve patient care through data collection in regional anaesthesia?	Not answered
Does regional anaesthesia increase the risk of harm from compartment syndrome?	Partly answered
What are the best methods for evaluating and comparing regional anaesthesia techniques?	Not answered
How can we improve patient involvement in decision making about regional anaesthesia?	Partly answered
What complications can occur from regional anaesthesia?	**FULLY ANSWERED**
How can regional anaesthesia be used most effectively in shoulder surgery?	Partly answered
What is the role of injection pressure monitoring in ultrasound guided regional anaesthesia?	Not answered
What are the risks and benefit of using a regional anaesthetic catheter technique?	Partly answered
How can access to regional anaesthesia be improved?	Not answered
How can we best manage pain as regional anaesthesia wears off?	Partly answered
What is the optimum duration of regional anaesthesia?	Not answered
What is the most effective dose and type of local anaesthetic for each regional anaesthesia technique?	Partly answered
Can new local anaesthetic agents or preparations help improve efficacy of regional anaesthesia?	Not answered
How does surgical infiltration with local anaesthetic compare with regional anaesthesia?	Partly answered
Do patients prefer surgery under general or regional anaesthesia?	Not answered
How can regional anaesthesia be used most effectively in genitourinary surgery?	Partly answered

How can regional anaesthesia be used most effectively for trauma patients?	Partly answered
Can regional anaesthesia improve short term recovery after surgery?	Partly answered
How do fascial plane blocks work?	Partly answered
What role does regional anaesthesia have in the management of patients with chronic pain?	Partly answered
How can we improve nomenclature in regional anaesthesia?	**FULLY ANSWERED**
What are the risks and benefits of mixing local anaesthetics?	Partly answered
