# Supplementary material for: Research priorities in regional anaesthesia: an international Delphi study
Source: Br J Anaesth. 2024 Mar 5;132(5):1041–8. doi: 10.1016/j.bja.2024.01.033 (PMC11103078; doi:10.1016/j.bja.2024.01.033)
Supplement: Multimedia component 5 [file mmc5.pdf]

Research priorities in regional anaesthesia: an international Delphi study

Supplementary material E – 53 Retained IQs

How can regional anaesthesia be used most effectively in abdominal surgery?
Can regional anaesthesia improve long term outcomes after surgery?
Which patients benefit most from regional anaesthesia?
What are the risks and benefits of regional anaesthesia for children?
How can regional anaesthesia be improved in low resource environments?
Can regional anaesthesia improve short term recovery after surgery?
How can the effectiveness of regional anaesthesia be improved?
How can patient experience of regional anaesthesia be improved?
How can regional anaesthesia be used most effectively for chest wall trauma?
How often do complications occur as a result of regional anaesthesia?
What is the safety profile of regional anaesthesia in patients with altered coagulation?
How can regional anaesthesia be used to optimise operating theatre efficiency?
How can regional anaesthesia be used most effectively in obstetric patients?
Can regional anaesthesia help to reduce the environmental impact of anaesthesia?
How can access to regional anaesthesia be improved?
How do fascial plane blocks work?
Can new local anaesthetic agents or preparations help improve efficacy of regional anaesthesia?
What are the risks and benefits of mixing local anaesthetics?
How can regional anaesthesia be used most effectively in breast surgery?
Which factors alter the duration of regional anaesthesia?
How can the safety profile of regional anaesthesia be improved?
How does sedation or general anaesthesia prior to block performance impact the safety of regional anaesthesia?
How can regional anaesthesia be used most effectively in hip surgery?
How should complications from regional anaesthesia be managed?
How can we improve patient care through data collection in regional anaesthesia?
What are the risks and benefit of using a regional anaesthetic catheter technique?
How can regional anaesthesia for emergency surgery be improved?
Does systemic absorption of local anaesthetic contribute to analgesia?
Can regional anaesthesia improve the management of pain after surgery?
How can regional anaesthesia be used most effectively for a fractured neck of femur?
How can regional anaesthesia be used most effectively in cardiothoracic surgery?
How can the safety of regional anaesthesia catheter techniques be ensured?
How can regional anaesthesia be used most effectively in shoulder surgery?
What is the optimum duration of regional anaesthesia?
Which, if any, sedation method should be used with regional anaesthesia?
How do the risks and benefits of regional anaesthesia change in different patient groups?
How can regional anaesthesia be used most effectively in knee surgery?
How can we improve the consent process for regional anaesthesia?
What is the optimal infusion regime for regional anaesthesia catheter techniques?

Is regional anaesthesia safer than general anaesthesia?
Does the safe dose of local anaesthetic vary between different patients and regional anaesthesia techniques?
How can regional anaesthesia be used most effectively for the intensive care patient?
How can regional anaesthesia be used most effectively in elbow surgery?
What are the best methods for evaluating and comparing regional anaesthesia techniques?
How can we improve patient involvement in decision making about regional anaesthesia?
How does surgical infiltration with local anaesthetic compare with regional anaesthesia?
How can regional anaesthesia for head and neck procedures be improved?
How can regional anaesthesia be used most effectively in spinal surgery?
Is regional anaesthesia more cost effective than alternatives?
Who should perform regional anaesthesia?
Does regional anaesthesia have an effect on cancer outcomes?
What is the most effective dose and type of local anaesthetic for each regional anaesthesia technique?
How can regional anaesthesia be used most effectively in genitourinary surgery?
